# Analogies in Oncology: Explanations Made Easier

**Published:** 2015-03-01

**Authors:** Wendy H. Vogel

There is little debate that it is overwhelming to receive a diagnosis of cancer. The intricacies of the disease process and the complexities of treatment are frequently difficult for patients to comprehend. Even experienced advanced practitioners (APs) may have difficulty understanding some concepts.

Advanced practitioners are often the main health-care providers to deliver education. This article will illustrate several favorite analogies utilized by oncology APs and other clinicians. The use of analogies may facilitate understanding of complex topics for both patients as well as health-care staff members (Azer, Guerrero, & Walsh, 2013; Edelen & Bell, 2011). One benefit of analogies is that they can give patients a mental picture to focus on while they navigate their disease. Patients may also find it easier to describe their disease process to concerned friends and family members when they are able to employ a clear analogy instead of unfamiliar, highly scientific terms.

## MULTIPLE MYELOMA: NATURAL HISTORY AND TREATMENT

**Beth Faiman, PhD(c), MSN, APN-BC, AOCN® Cleveland Clinic Taussig Cancer Institute**

Multiple myeloma (MM) is a clonal disorder of the plasma cells. However, the abnormal plasma cells will change over time (Keats et al., 2012). A basic tenet in MM is to treat the cancer as a chronic, incurable condition to be managed with ongoing therapy, adapting treatment as the disease changes. Immunoglobulins are created by plasma cells, yet the immunoglobulins in MM can be thought of as a bully on the playground. The first "bully" (the first abnormal immunoglobulins) can be eradicated with chemotherapy about 80% of the time with the A team (the first treatment), regardless of the agent used to treat the MM. This bully will disappear, but after a few months or years, another bully (immunoglobulins with new changes) will reemerge. Then we will have to use the B team (second-line treatment) to go after this next bully. Unfortunately, the bullies get smarter and meaner until our treatment is no longer effective.

This analogy could be quite useful for educating new APs or students about MM. It illustrates the natural history of the disease, the need for continual vigilance for new "bullies," and the need for successive treatments.

## IMMUNOMODULATING AGENTS: MECHANISM OF ACTION

**Beth Faiman, PhD(c), MSN, APN-BC, AOCN® Cleveland Clinic Taussig Cancer Institute**

When describing the mechanism of action of immunomodulating agents (lenalidomide [Revlimid], thalidomide [Thalomid], pomalidomide [Pomalyst]), a garden analogy and the concept of "weed and feed" may be employed (Zhu, Kortuem, & Stewart, 2013). This method is useful to describe the rationale for ongoing continuous therapy (maintenance therapy) to patients. The bone marrow may be pictured as a garden or lawn. The soil on top looks beautiful. However, there are weeds lurking underneath the ground that cannot be seen. The weeds represent clonal plasma cells: clones of the normal cells in the bone marrow.

Treatment of the MM will get rid of the weeds. Continuous therapy is weed and feed for the bone marrow: The treatment kills the weeds by eliminating oxygen, blood supply, and other things the cancer cells need to grow. By eliminating the weeds, the healthy, normal blood cells can then grow (white blood cells, red blood cells, and platelets). This analogy may be useful for both patients and those in the health-care field.

## TREATMENTS AND THE HEALTH-CARE TEAM

**Wendy J. Smith, ACNP, AOCN®**

**Memorial/University of Colorado**

Describing treatment effects on a malignancy can be difficult. When discussing how treatments affect cell receptors, consider having the patient picture a golf ball, with all the little ridges around the surface of the golf ball representing receptors on a cell’s surface. If you were to roll the golf ball through a puddle of paint, some of those receptors would pick up the paint (the targeted treatment).

The difference between local and systemic treatments may be explained by imagining cancer as a dandelion. If you pull it up by the roots before it gets those fuzzy seeds (local treatment), you kill the plant. But if the dandelion gets those fuzzy seeds that blow off, you may see where some of those fuzzy seeds go; unfortunately, there are other fuzzy seeds out there that cannot be seen. These seeds have the potential for landing somewhere else in the body, and they might grow (metastatic disease). Systemic treatment is like spraying the whole yard with weed killer (this is like systemic treatment). Unfortunately, the weed killer can also affect normal cells, causing side effects.

A team analogy can be useful to explain various roles in cancer treatment: the role of the patient, the health-care provider, and others. Treating cancer is a team effort. It can be explained with this analogy: Cancer treatment is not something we do TO the patient; it is something we do WITH the patient. The oncologist is the head coach, APs are the assistant coaches, and the patient is the quarterback. The oncologist decides the game plan. The APs help coach the patient to carry out the head coach’s plays. Once into the game, if the chosen plan (such as the antiemetic regimen) isn’t working down on the field, the quarterback (the patient) lets the coaches know so that the plan can be revised. Communication between teammates and coaches is essential. We don’t send a quarterback (patient) onto the field into a rough game without knowing or understanding the game plan. This analogy may facilitate communication not only between patients and the health-care team, but also between members of the health-care team.

**Kathy Sharp, MSN, FNP-BC, AOCNP®, CCD**

**Wellmont Cancer Institute**

When describing treatment modalities to patients, this analogy may prove useful: Killing cancer is like killing bears in the woods. First, you remove any bears you can see. This may be accomplished with surgery—the physical removal of a cancer. However, some bears may be hiding, so often the next step is to spray the underbrush with "bear-killer spray." This represents adjuvant systemic treatment, such as chemotherapy. This bear-killer spray works to destroy any bears that cannot be seen. But some bears can climb trees and hide in the treetops. These bears can be removed with a controlled fire, which represents radiation therapy.

The concept of a stage 0 cancer may be difficult for the AP to explain to patients. This analogy is useful in explaining the rationale for adjuvant hormonal therapy to stage 0 breast cancer patients. Picture a bear in hibernation (the analogy of a stage 0 cell growth). Bears in hibernation are usually hidden in caves. They do not consume food and get no outside air, so they cannot be killed by starvation, shotguns (systemic treatments), or fire (radiation) because the bear is protected in the cave. This is much like a cancer cell that cannot be seen or killed while in stage 0. If the conditions are right, the female bear has cubs while in hibernation. When she wakes up and comes out of the den, there can be two or three bears instead of one. When a cancer cell comes out of stage 0 growth, it begins to reproduce as well. If the food supply is low and the bear cannot put on enough weight to last her during hibernation, she cannot get pregnant. In the case of breast cancer, giving adjuvant treatment with hormone-blocking drugs such as an aromatase inhibitor essentially cuts off the food supply so the cancer cell (like the bear) cannot reproduce.

## TARGETED THERAPY

**Wendy Vogel, MSN, FNP, AOCNP®**

**Wellmont Cancer Institute**

The difference between chemotherapy and targeted therapy may be described as the difference between a shotgun and a laser gun. The shotgun (chemotherapy) is effective but can cause a lot more damage, i.e., pellet scatter, in many areas of the body (systemic side effects). Biotherapy is like a laser gun: more focused, with less, but very specific, damage (side effects; Bourdeanu & Luu, 2014). Biotherapy targets the area the laser gun is "aimed at."

The mechanism of action of some targeted therapies may also be explained by the analogy of a singles bar. The singles bar is represented by a cancer cell and receptors on the cancer cell. The receptor site is looking to "hook up" with its favorite dream lover (or ligand) for a romantic interlude. The romantic interlude is the process of hooking up: the receptor site and the ligand join (dimerization), causing downstream signaling to "turn on" (activation of) the pathways (such as the kinase pathway) so that the cell will replicate, metastasize, or lose normal apoptosis.

The targeted therapy can be visualized as "Mama," who is going to stop the romantic interlude. If Mama catches the ligand in the parking lot and keeps it from hooking up with a growth factor receptor, that is an extracellular action, which is how antiangiogenic agents such as bevacizumab (Avastin) work. If Mama stops the action at the singles bar door—working at the cell surface to keep the growth factor receptor and its ligand from joining (dimerization)—that is how drugs like EGFR and HER2 inhibitors work. And if Mama has to go into the singles bar (into the cell) in order to stop the romantic interlude, then that is intracellular activity, where small-molecule drugs like mTOR inhibitors work, stopping the downstream signaling process.

## EXPLAINING HEMATOPOIESIS

**Kathy Sharp, MSN, FNP-BC, AOCNP®, CCD**

**Wellmont Cancer Institute**

Patients do not always have a good understanding of where their blood cells come from. This analogy may be helpful when educating about anemias and their etiologies. Making blood (hematopoiesis) is like making a cake. In order to make a cake one must have the basic ingredients: flour, sugar, and eggs. But just having the ingredients does not make a cake. Even if all the ingredients are present, they must be mixed correctly and baked correctly. If the mixer is broken or the oven isn’t working, it is difficult to produce a cake. Likewise, to make blood, the bone marrow must have iron, B_12_, folate, and erythropoietin. If the bone marrow (the oven) is not functional, blood may be made but not released into the bloodstream or not made at all.

Anemia could be a deficiency problem—the lack of iron or B12 or folate—that can be described as similar to not having the needed ingredients to bake a cake. But anemia (or thrombocytopenia) could be a destruction problem: The spleen is retaining the blood cells or the blood cells are being destroyed once they have left the bone marrow. This could be compared to making a perfect cake, but then having someone (the spleen) hide it so it cannot be eaten (utilized). The perfectly made cake might also be destroyed if it is wrongly perceived to be poisonous. This is a simplification of an autoimmune problem in which the body’s immune system perceives the blood cells to be "foreign" to the body and destroys them.

**Aurora Roasa, RN, BSN**

**University of Maryland**

**Greenebaum Cancer Center**

The concept of hematopoiesis may be complicated to explain to both patients and students. The bone marrow produces blood, likening it to the "factory." If the factory becomes defective or dirty, it must be shut down for a general cleaning, as likened to a high-dose chemotherapy regimen that "cleanses" the marrow. This process can be compared to a clean blue shirt. Imagine a bright red wine stain on it (leukemia). It needs to be washed with the strongest detergent that contains bleach. Unfortunately, you can’t just wash the spot—you need to wash the whole shirt (treatment with systemic chemo). During the process of "systemic" cleaning, the blue part of the shirt gets affected by the bleach-containing detergent (chemo), just like the various cells (platelets, red blood cells, and white blood cells) are "washed away" and the body has to wait for the new cells to regenerate. Transplantation can be likened to adding a new dye color to the bleached shirt so that it can be presentable (functional) again.

**Nancy Corbitt, BSN, RN, OCN®, CRNI**

**University of Maryland**

**Greenebaum Cancer Center**

Visual analogies may be useful as well. When teaching patients about chemotherapy’s effect on the bone marrow, a dry erase board might be utilized to draw a picture of the bone marrow. During discussion of the different cells of the bone marrow and the chemotherapy effect on the cells, chemotherapy can be described as an eraser that wipes all the cells out until the bone marrow regenerates new cells. Different colored markers might be utilized for the different types of blood cells.

## YOUR FAVORITE ANALOGIES

Analogies can be invaluable for patient education. What analogies do you use in your practice? Discuss it with your colleagues, and e-mail us some of your favorite explanations at editor@advancedpractitioner.com. The editors will contact you if they plan to use your analogy in a future *JADPRO* article or website feature.
